# Clinical efficacy and hemodynamic effects of levosimendan in cardiac surgery patients after surgery

**DOI:** 10.1186/s13019-024-03316-3

**Published:** 2025-01-07

**Authors:** Meiling Zhao, Yunfeng Hou, Meng Yuan, Shuang Ma, Yifeng Yue

**Affiliations:** 1 Department of Critical Care Medicine, Zibo Central Hospital, Zibo, 255000 China; 2 Department of Critical Care Medicine, The First Affiliated Hospital of Shandong First Medical University, JiNan, 250014 China; 3 Department of Anesthesiology, Zibo Central Hospital, No.10 Shanghai Road, Zhangdian District, Zibo City, 255000 Shandong Province China

**Keywords:** Levosimendan, Cardiac surgery, Acute cardiac insufficiency, Clinical efficacy, Hemodynamics

## Abstract

**Objective:**

To investigate the therapeutic effect of levosimendan on hemodynamics in patients undergoing major cardiac surgery and presenting with acute postoperative heart failure.

**Methods:**

The subjects of the study were 160 patients with severe cardiac conditions who underwent surgery and had acute heart failure. Eighty cases each were assigned to the research and control groups using a random number table. Document the general patient data for each of the two groups; compare the clinical outcomes of the two groups. The hemodynamic states of the two groups were compared both before and after therapy. 48 h after surgery, echocardiography was performed in both groups to determine cardiac function. 48 h after surgery, *N*-terminal pro-brain *B*-type natriuretic peptide (NT-Pro-BNP) levels were compared between the two groups.

**Results:**

The overall effective rate was significantly higher in the research group (92.5%) compared to the control group (76.25%, *P* < 0.05). Post-treatment, the research group demonstrated a significant reduction in CVP (9.25 ± 2.11 cmH2O vs. 11.36 ± 3.08 cmH2O, *P* < 0.001), heart rate (100.30 ± 8.69 bpm vs. 105.74 ± 7.69 bpm, *P* < 0.001), and lactic acid levels (1.68 ± 0.59 mmol/L vs. 2.69 ± 0.55 mmol/L, *P* < 0.001). The research group also showed improvements in SBP (117.23 ± 8.74 mmHg vs. 113.25 ± 7.55 mmHg, *P* = 0.002) and urine output (4.21 ± 1.76 mL/kg/h vs. 3.65 ± 1.23 mL/kg/h, *P* = 0.021). Cardiac function indicators 48 h after surgery indicated a higher LVEF (55.21 ± 8.04% vs. 47.18 ± 6.60%, *P* < 0.001) and lower LVEDVi and LVESVi in the research group (*P* < 0.001 for both). NT-Pro-BNP levels were significantly lower in the research group (6010.19 ± 1208.52 pg/mL vs. 9663.21 ± 2391.34 pg/mL, *P* < 0.001). The incidence of complications was lower in the research group (5% vs. 22.5%, *P* = 0.001).

**Conclusion:**

Cardiac surgery patients are prone to complications with acute heart failure after surgery. Treatment with levosimendan can significantly improve clinical efficacy and reduce complications. It can also effectively improve patients' cardiac function and promote hemodynamic stability.

## Introduction

Acute postoperative heart failure is a serious complication that affects a significant proportion of patients undergoing major cardiac surgery. It is estimated that up to 20% of patients may develop acute heart failure following cardiac surgery, contributing to increased morbidity and mortality rates. This condition often necessitates immediate medical intervention due to the high risk of poor outcomes, including prolonged hospital stays and increased healthcare costs [19, 10–13]. The standard treatment for acute postoperative heart failure typically involves the use of vasoactive drugs to stabilize hemodynamics and support cardiac function [9]. Commonly used agents include inotropes such as dopamine, dobutamine, and epinephrine, which enhance myocardial contractility and improve cardiac output [11, 25]. However, long-term and high-dose use of vasoactive drugs may bring some serious side effects, including cardiac arrhythmia, myocardial damage, and poor organ perfusion, which affects the recovery rate of patients, and is not conducive to prognosis [5, 14, 21]. Therefore, the exploration of therapeutic drugs with fewer side effects and significant efficacy has become a hot research topic, which is of great significance for the intervention of acute heart failure in patients with severe cardiac conditions undergoing cardiac surgery.

Levosimendan is a calcium ion sensitizer and a novel cardiac stimulant. It increases cardiac output by enhancing myocardial cell contractility while dilating peripheral blood vessels and reducing cardiac afterload. It is currently widely used to treat heart failure [6, 15, 23]. Compared with other positive inotropic drugs, the study [3, 27] found that levosimendan has a significant effect in the treatment of acute heart failure with fewer side effects, which is worthy of clinical promotion. Therefore, to guide the therapeutic use of levosimendan, this research aimed to investigate the clinical efficacy of levosimendan and its effects on hemodynamics in patients who underwent major cardiac surgery and had immediate postoperative heart failure at the same time.

## Method and materials

### Research subject

The research participants were patients with severe cardiac conditions who underwent surgery for acute heart failure in the Department of Cardiac Surgery. Inclusion criteria: ① All selected patients met New York Heart Association cardiac function classification levels III-IV; ② Left ventricular ejection fraction (LVEF) < 45%; ③ Age ≥ 18 years; ④ Informed consent was signed by patients or their families. It is important to note that the clinical implications of NYHA III-IV classification and LVEF < 0.45 vary between different pathologies. For example, the hemodynamic impact is different for patients with mitral valve disease versus those with aortic valve disease. The patients were accordingly stratified into groups reflecting these differences. Exclusion criteria: ① Individuals suffering from multi-organ disease, including kidney and liver; ② Patients with an implanted pacemaker; ③ Patients with allergic reactions after drug administration; ④ Patients were automatically discharged from the hospital during the experiment. The hospital ethics committee of Zibo Central Hospital reviewed and approved this project (No.202112006). This study has been performed in accordance with the Declaration of Helsinki.

The study cohort included a heterogeneous population of patients with coronary artery disease, valve disease, and congenital heart diseases. Each of these conditions was categorized into specific subgroups, with the number of patients in each subgroup clearly defined. This stratification aimed to provide a clearer understanding of the patient demographics and ensure appropriate analysis based on disease types.

### Method

#### Therapeutic method

The randomization process in this study was conducted using a computer-generated random number table. This method was selected to ensure an unbiased and scientifically rigorous allocation of participants to either the research group or the control group. To ensure the baseline characteristics of the two groups were comparable in this study, we implemented a stratified randomization process combined with block randomization. The stratification was based on key variables such as age, gender, and severity of heart failure, which are known to influence the outcomes of interest.

Patients in the control team received standard treatment, including venous access, oxygen inhalation, and ECG monitoring. Depending on the specific condition of the patients, they received basic medications such as traditional cardiac reinforcement, diuresis, and vasodilatation.

The research team patients were administered an initial loading dose of levosimendan at 12 µg per kilogram (μg/kg) over a 10-min period. This was followed by a continuous intravenous infusion. After the loading dose, levosimendan was infused at a rate of 0.1 µg per kilogram per minute (μg/kg/min). The infusion was maintained for 24 h to ensure sustained hemodynamic support. The levosimendan was prepared by diluting 5 ml (ml) of levosimendan (2.5 mg/ml) in 45 ml of 5% glucose solution, resulting in a total volume of 50 ml. This solution was then administered using an infusion pump to maintain the precise infusion rate. The protocol was closely monitored, and adjustments were made if necessary based on the patient's hemodynamic response and clinical condition. Throughout the infusion, patients were continuously monitored for vital signs, including heart rate, blood pressure, and central venous pressure, as well as for any signs of adverse effects. Regular blood tests were conducted to measure lactic acid levels and other relevant biochemical markers. During surgery, myocardial protection was ensured through the use of cardioplegia. This method helps protect the heart from ischemic injury by temporarily stopping the heart and reducing metabolic demand during the procedure.

Patients were enrolled based on inclusion and exclusion criteria, and randomization was conducted using a computer-generated random number table. Block randomization and stratification were employed based on key factors such as age, gender, and heart failure severity to ensure balanced group assignments.

#### Observation indicators

1. General information was compared between the two groups. General information about the patients, such as gender, age, body mass index, coronary heart disease, hypertension, diabetes, smoking, alcohol consumption, NYHA classification, and type of surgery, was collected from medical records and electronic medical records.
2. Clinical efficacy was compared between the two groups. Evaluation criteria for clinical efficacy: marked efficacy: improvement in NYHA classification was NYHA grade 2 or higher, and clinical symptoms and signs were significantly improved. Effective: Improvement in NYHA grade ≥ 1, improvement in clinical symptoms and signs; Ineffective: No significant change or worsening in NYHA grade, clinical symptoms and signs. Overall effectiveness rate = (significantly effective + effective)/total number of cases × 100%. The primary endpoint of this study was improvement in cardiac function as measured by LVEF at 48 h post-surgery. Secondary endpoints included changes in NT-proBNP levels, hemodynamic parameters, and the incidence of complications. Appropriate adjustments were made for multiple endpoints in the statistical analysis.
3. Before and after therapy, the hemodynamic status of the two groups was compared. Heart rate, urine volume, systolic blood pressure, central venous pressure, and lactic acid levels were monitored.
4. Cardiac function 48 h after surgery was compared between the two groups. Left ventricular end-diastolic volume index (LVEDVi), left ventricular end-systolic volume index (LVESVi) and LVEF were determined by echocardiography.
5. 48 h after surgery, *N*-terminal natriuretic peptide type *B* (NT-Pro-BNP) levels were compared in the two groups. Collect fasting venous blood, let it stand, centrifuge it, and take the supernatant for detection by enzyme-linked immunosorbent assay (ELISA).
6. A comparison of the incidence of various problems such as arrhythmia, low cardiac output, renal failure, etc. was made between the two groups. Total incidence = (low cardiac output syndrome + arrhythmia + renal failure)/total number of cases × 100%.

#### Assessment

Hemodynamic parameters, including central venous pressure (CVP), systolic blood pressure (SBP), heart rate (HR), urine output, and lactic acid levels, were measured at baseline (before intervention) and at regular intervals during and after the levosimendan infusion. CVP was measured using a central venous catheter connected to a pressure transducer calibrated to zero at the midaxillary line. Measurements were taken in millimeters of mercury (mmHg). SBP was continuously monitored using an arterial line connected to a pressure transducer, with readings displayed on a bedside monitor. SBP was recorded in millimeters of mercury (mmHg). HR was continuously monitored using standard ECG leads, with the average heart rate over a 1-min period recorded in beats per minute (bpm). Urine output was measured hourly using a Foley catheter with a graduated collection bag. The volume was recorded in milliliters per kilogram per hour (mL/kg/h). Blood samples were drawn from an arterial line, and lactic acid levels were measured using a point-of-care blood gas analyzer. Results were recorded in millimoles per liter (mmol/L).

Cardiac function was assessed using transthoracic echocardiography (TTE) 48 h postoperatively. The key parameters evaluated included left ventricular ejection fraction (LVEF), left ventricular end-diastolic volume index (LVEDVi), and left ventricular end-systolic volume index (LVESVi). A standard transthoracic echocardiography machine (e.g., GE Vivid S70 or Philips EPIQ 7) was used. LVEF was calculated using the Simpson’s biplane method, and LVEDVi and LVESVi were indexed to body surface area. Measurements were performed by a cardiologist blinded to group assignments to reduce bias. NT-Pro-BNP levels were quantified using a high-sensitivity enzyme-linked immunosorbent assay (ELISA) kit (e.g., Roche Elecsys). Results were expressed in picograms per milliliter (pg/mL). Continuous ECG monitoring was used to detect and document arrhythmias, with events classified according to the American Heart Association (AHA) guidelines. Renal function was assessed using serum creatinine levels and urine output. Acute kidney injury was defined according to the kidney disease: improving global outcomes (KDIGO) criteria. This was defined as the need for mechanical circulatory support or inotropic therapy beyond standard care, based on clinical signs such as hypotension, decreased urine output, and poor peripheral perfusion.

### Statistical analysis

SPSS 21.0 software was used for the statistical analysis. Measurement indicators corresponding to homogeneity of variance and normal distribution were recorded as mean ± standard deviation, while count indicators were recorded as [number of cases (percentage)]. Independent samples *t*-test and *χ*2 test were used for comparison between groups (*P* < 0.05 indicates a statistically meaningful disparity. Sample size estimation was based on a power calculation. We assumed a significant difference in clinical outcomes between the research and control groups, with a power of 80% and a significance level of 0.05. Based on these assumptions, a minimum sample size of 80 patients per group was determined to be sufficient.

## Results

### General data contrast between the two groups

The research participants were 160 patients with severe cardiac conditions who underwent surgery for acute heart failure in the Department of Cardiac Surgery of our hospital between October 2022 and July 2023. The sample consisted of 77 women and 83 men aged between 40 and 72 years, with a mean age of (55.69 ± 6.40) years. The baseline characteristics of the participants in the study and control groups were analyzed to ensure comparability. No significant differences were observed between the two groups in terms of age, gender, body mass index (BMI), prevalence of comorbid conditions (such as coronary artery disease, hypertension, and diabetes), smoking and alcohol consumption habits, NYHA classification, or type of surgery (*P* > 0.05 for all variables), indicating that the groups were well-matched at baseline (Table 1).

**Table 1:** Comparison of general data between the two groups

General information	Study team (*n* = 80)	Control team (*n* = 80)	χ^2^/t	P
Sex (n, %)			0.626	0.429
Man	44 (55.00)	39 (48.75)		
Woman	36 (45.00)	41 (51.25)		
Age (years)	55.21 ± 6.39	56.18 ± 6.43	0.950	0.343
BMI (kg/m^2^)	21.02 ± 0.96	20.89 ± 1.02	0.829	0.408
Coronary heart disease (n, %)			0.102	0.749
Yes	33 (41.25)	35 (43.75)		
No	47 (58.75)	45 (56.25)		
Hypertensive (n, %)			0.404	0.525
Yes	38 (47.50)	34 (42.50)		
No	42 (52.50)	46 (57.50)		
Diabetes (n, %)			0.236	0.627
Yes	30 (37.50)	33 (41.25)		
No	50 (62.50)	47 (58.75)		
Cigarette smoking (n, %)			0.100	0.752
Yes	40 (50.00)	38 (47.50)		
No	40 (50.00)	42 (52.50)		
Drinking wine (n, %)			0.100	0.752
Yes	43 (53.75)	41 (51.25)		
No	37 (46.25)	39 (48.75)		
NYHA Classification (n, %)			0.129	0.719
Level III	58 (72.50)	60 (75.00)		
Level IV	22 (27.50)	20 (25.00)		
Type of surgery (n, %)			0.808	0.668
Valve replacement	62 (77.50)	59 (73.75)		
Coronary bypass operation	10 (12.50)	14 (17.50)		
Correction of congenital heart disease	8 (10.00)	7 (8.75)		

### Comparing the two groups’ clinical outcomes

**Fig. 1:**
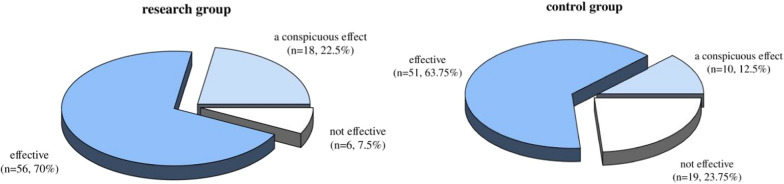
Comparing the two groups’ clinical outcomes

The overall clinical efficacy was significantly higher in the research group compared to the control group. Specifically, 92.5% (*n* = 74) of patients in the research group exhibited marked or effective improvement in NYHA classification, compared to 76.25% (*n* = 61) in the control group (*P* = 0.005). The proportion of patients with marked improvement was 22.5% (*n* = 18) in the research group, as opposed to 12.5% (*n* = 10) in the control group (Table 2; Fig. 1). These findings suggest that levosimendan significantly enhances clinical outcomes in patients with acute postoperative heart failure.

**Table 2:** Comparing the two groups’ clinical outcomes (n, %)

Evaluations	Study team (*n* = 80)	Control team (*n* = 80)	χ^2^	P
Marked effectiveness	18 (22.50)	10 (12.50)	–	–
Effective	56 (70.00)	51 (63.75)	–	–
Ineffective	6 (7.50)	19 (23.75)	–	–
Total effective rate	74 (92.50)	61 (76.25)	8.012	0.005

### Hemodynamic contrast between the two groups before and after therapy

**Fig. 2:**
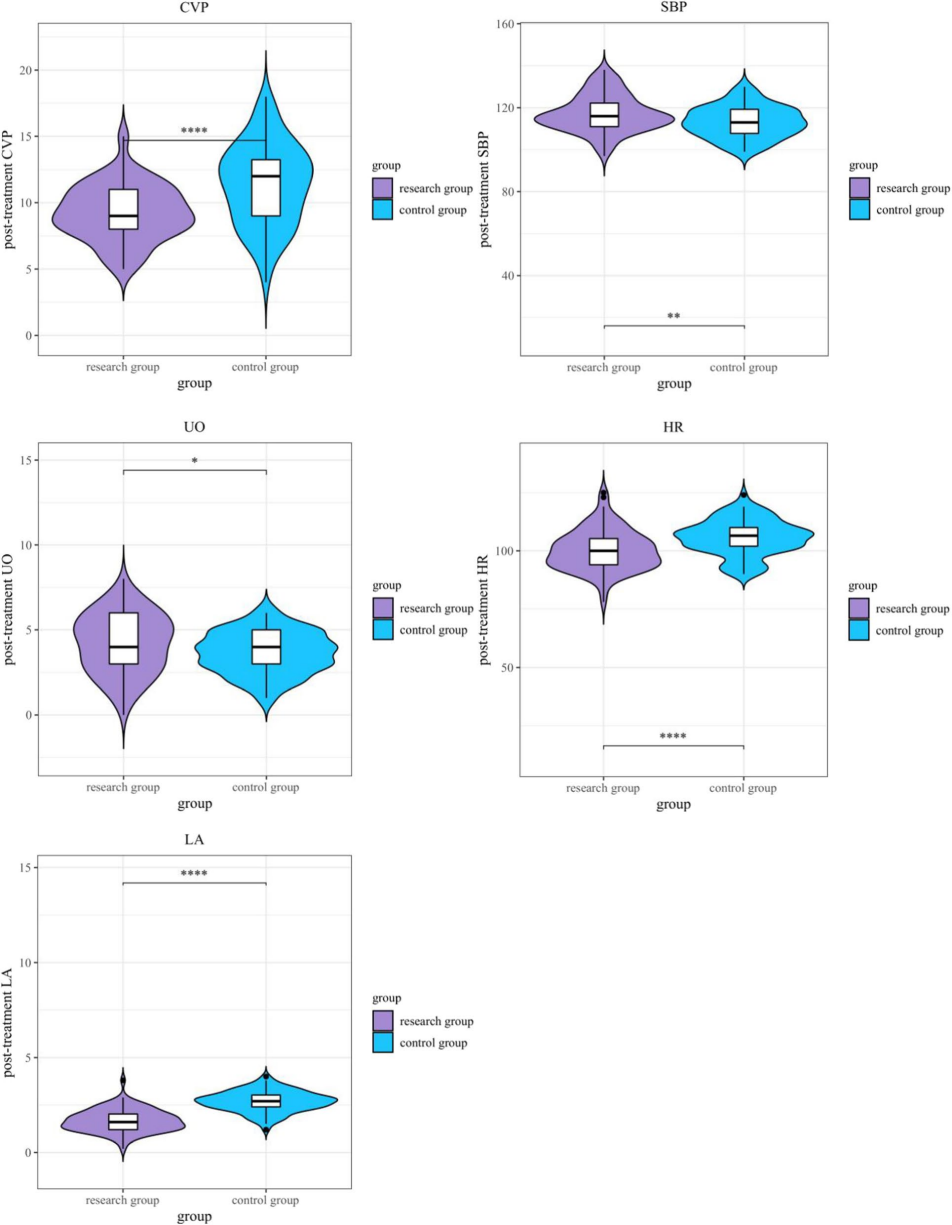
Contrast of the two groups’ post-treatment CVP, Systolic blood pressure, urine volume, heart rate, and lactic acid level

The analysis of hemodynamic parameters before and after treatment revealed significant improvements in the research group. Post-treatment central venous pressure (CVP) was significantly lower in the research group (9.25 ± 2.11 cmH_2_O) compared to the control group (11.36 ± 3.08 cmH_2_O, *P* < 0.001). Systolic blood pressure (SBP) was significantly higher in the research group post-treatment (117.23 ± 8.74 mmHg) compared to the control group (113.25 ± 7.55 mmHg, *P* = 0.002). Additionally, heart rate (HR) was significantly reduced in the research group (100.30 ± 8.69 bpm) compared to the control group (105.74 ± 7.69 bpm, *P* < 0.001). Urine output was higher in the research group (4.21 ± 1.76 mL/kg/h) than in the control group (3.65 ± 1.23 mL/kg/h, *P* = 0.021), and lactic acid levels were lower in the research group (1.68 ± 0.59 mmol/L) compared to the control group (2.69 ± 0.55 mmol/L, *P* < 0.001) (Table 3; Fig. 2). These results demonstrate that levosimendan contributes to improved hemodynamic stability in this patient population.

**Table 3:** Before and after therapy, the central venous pressure, systolic blood pressure, urine volume, heart rate and lactic acid levels of the two groups were compared ((x ± s)

Hemodynamics		Study team (n = 80)	Control team (*n* = 80)	*t*	*P*
Central venous pressure (cmH^2^O)	Pre-treatment	18.99 ± 4.72	18.90 ± 4.72	0.117	0.907
	Post-treatment	9.25 ± 2.11	11.36 ± 3.08	5.060	< 0.001
Systolic blood pressure (mmHg)	Pre-treatment	106.38 ± 10.20	105.30 ± 10.61	0.653	0.515
	Post-treatment	117.23 ± 8.74	113.25 ± 7.55	3.077	0.002
Urine volume [mL/(kg h)]	Pre-treatment	1.63 ± 0.83	1.65 ± 0.87	0.185	0.853
	Post-treatment	4.21 ± 1.76	3.65 ± 1.23	2.339	0.021
Heart rate (beats /min)	Pre-treatment	115.28 ± 12.37	116.38 ± 11.99	0.571	0.569
	Post-treatment	100.30 ± 8.69	105.74 ± 7.69	4.192	< 0.001
Lactic acid (mmol/L)	Pre-treatment	7.27 ± 2.11	7.19 ± 2.06	0.235	0.814
	Post-treatment	1.68 ± 0.59	2.69 ± 0.55	11.164	< 0.001

### Contrast of cardiac function in the two groups 48 h after operation

**Fig. 3:**
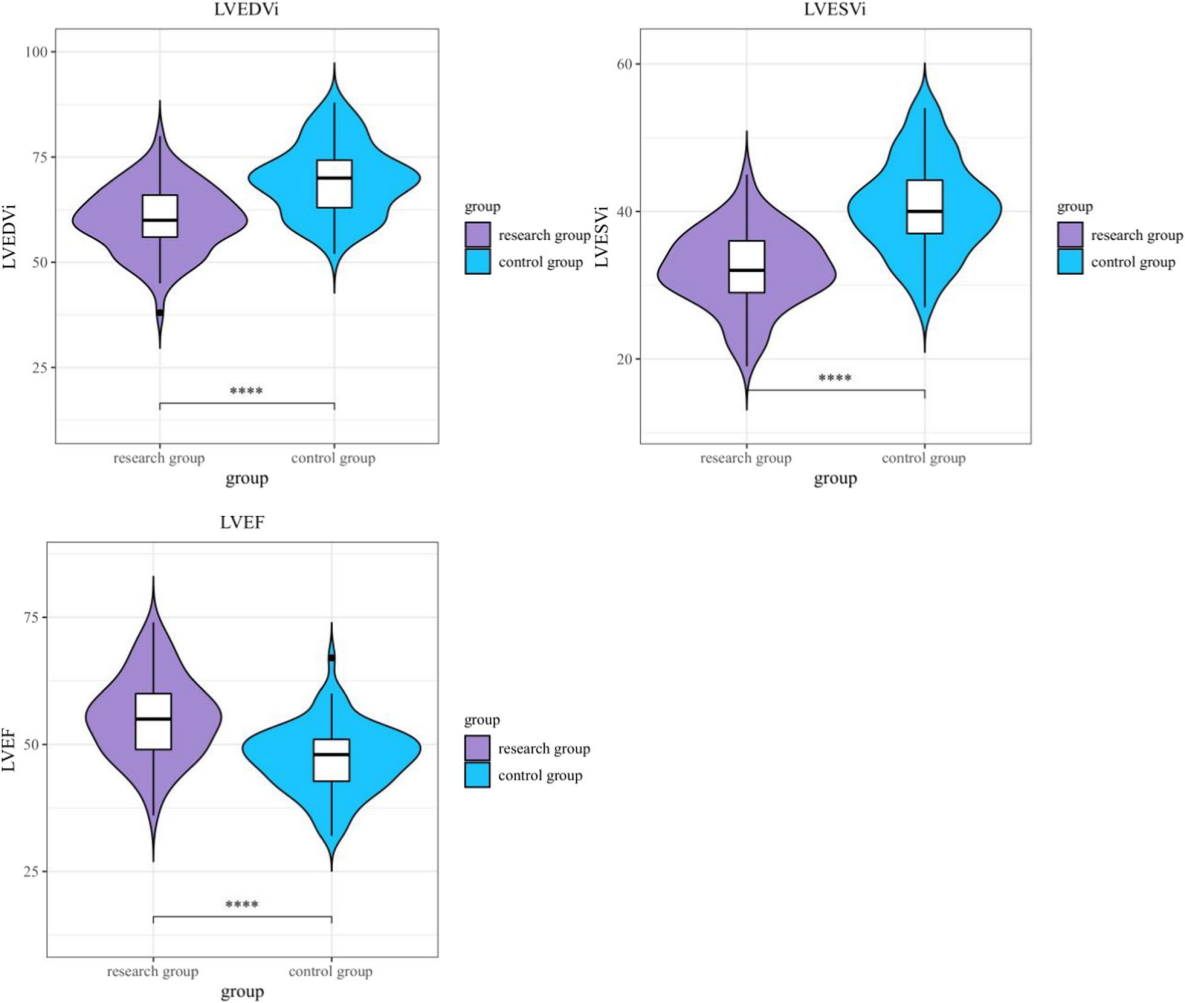
Contrast of the two groups’ LVEDVi, LVESVi, and LVEF values 48 h following Surgery

Cardiac function was evaluated 48 h postoperatively using echocardiography. The left ventricular ejection fraction (LVEF) was significantly higher in the research group (55.21 ± 8.04%) compared to the control group (47.18 ± 6.60%, *P* < 0.001). Additionally, the left ventricular end-diastolic volume index (LVEDVi) and left ventricular end-systolic volume index (LVESVi) were significantly lower in the research group (60.24 ± 7.87 mL/m^2^ and 32.18 ± 5.22 mL/m^2^, respectively) than in the control group (69.55 ± 8.28 mL/m^2^ and 40.64 ± 6.32 mL/m^2^, *P* < 0.001 for both) (Table 4; Fig. 3). These findings indicate that levosimendan effectively improves left ventricular function in patients with acute postoperative heart failure.

**Table 4:** Contrast of the two groups’ LVEDVi, LVESVi, and LVEF values 48 h following surgery ($\overline{x} \pm s$)

Cardiac function	Study team (*n* = 80)	Control team(*n* = 80)	*t*	*P*
LVEDVi (mL/m2)	60.24 ± 7.87	69.55 ± 8.28	7.293	< 0.001
LVESVi (mL/m2)	32.18 ± 5.22	40.64 ± 6.32	9.229	< 0.001
LVEF (%)	55.21 ± 8.04	47.18 ± 6.60	6.912	< 0.001

### NT-Pro-BNP levels

**Fig. 4:**
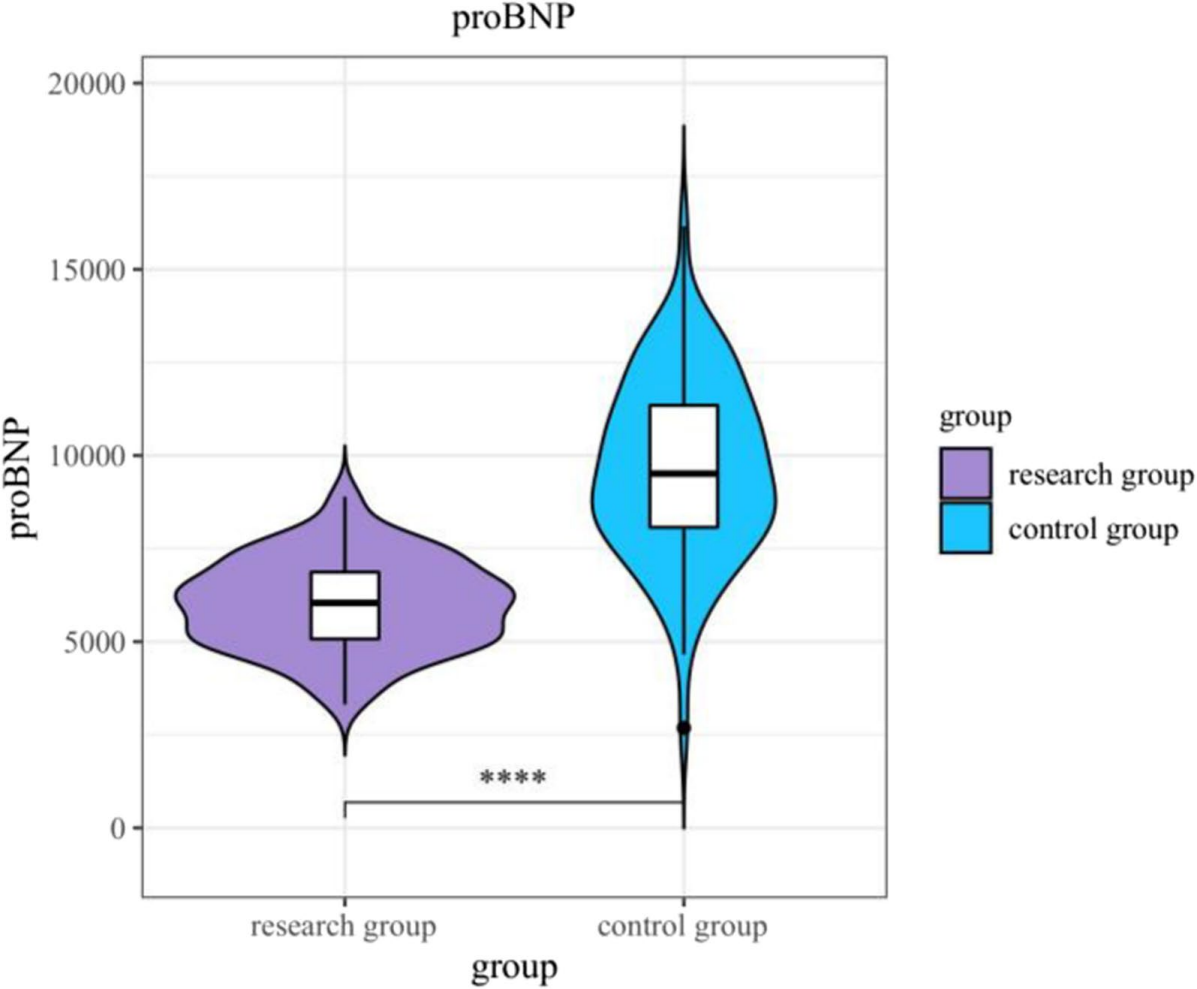
Comparison of NT-Pro-BNP levels between the two groups

NT-Pro-BNP levels, measured 48 h after surgery, were significantly lower in the research group (6010.19 ± 1208.52 pg/mL) compared to the control group (9663.21 ± 2391.34 pg/mL, *P* < 0.001) (Table 5; Fig. 4). This significant reduction in NT-Pro-BNP levels suggests that levosimendan treatment is associated with improved cardiac function and reduced ventricular stress.

**Table 5:** Contrast of NT-Pro-BNP levels between the two groups ($\overline{x} \pm s$)

Index	Study team (*n* = 80)	Control team (*n* = 80)	*t*	*P*
NT-Pro-BNP (pg/mL)	6010.19 ± 1208.52	9663.21 ± 2391.34	12.195	< 0.001

### Contrast of complications between the two groups

**Fig. 5:**
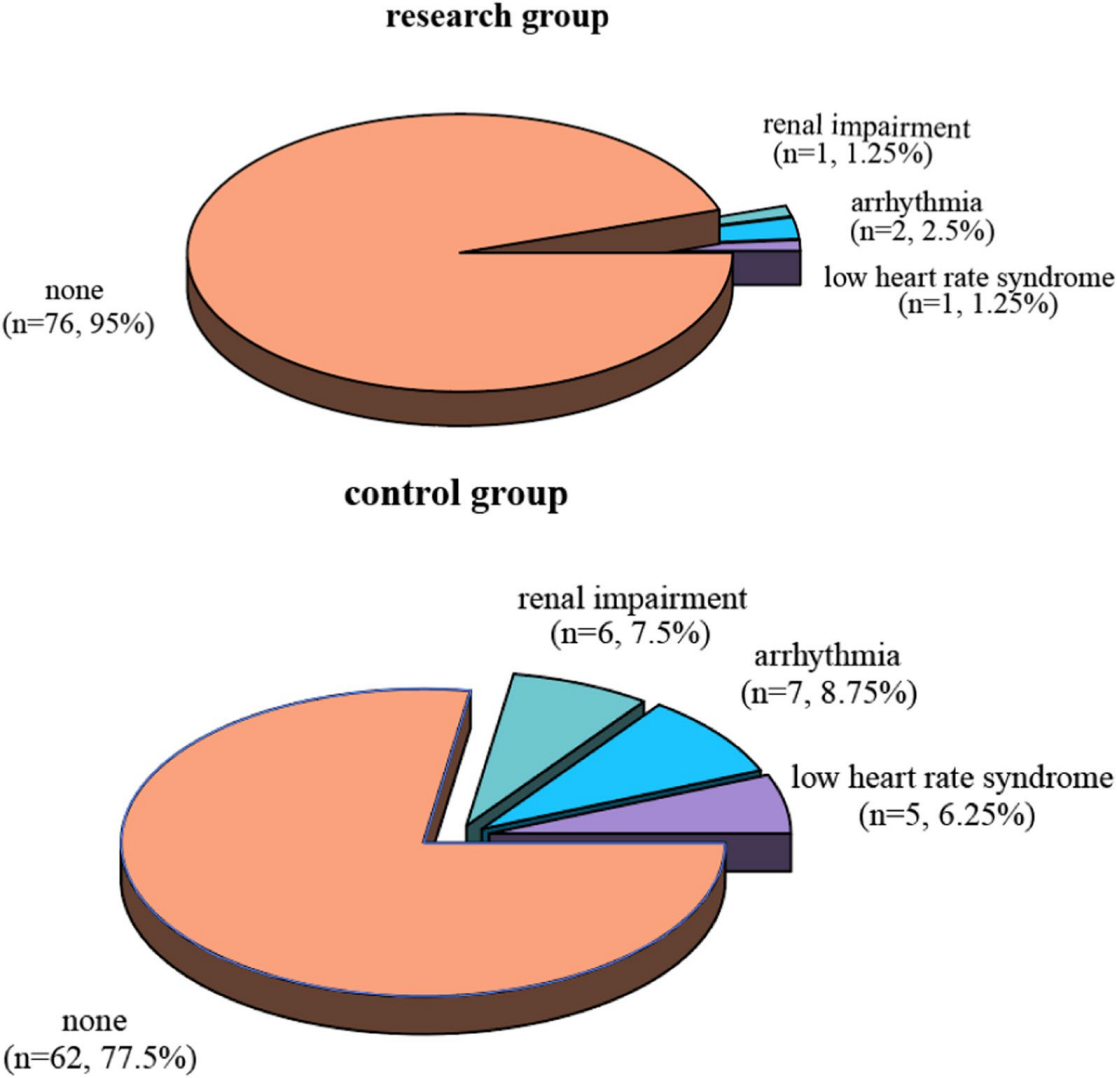
Contrast of complications between the two groups

The incidence of postoperative complications was significantly lower in the research group compared to the control group. The overall complication rate was 5.00% (*n* = 4) in the research group versus 22.50% (*n* = 18) in the control group (*P* = 0.001). Specifically, the incidence of low cardiac output syndrome, arrhythmias, and renal impairment was lower in the research group (1.25%, 2.50%, and 1.25%, respectively) compared to the control group (6.25%, 8.75%, and 7.50%, respectively) (Table 6; Fig. 5). These results indicate that levosimendan not only improves clinical outcomes but also reduces the risk of postoperative complications.

**Table 6:** Contrast of complications between the two groups (n, %)

Complication	Study team (*n* = 80)	Control team (*n* = 80)	χ^2^	P
Low cardiac output syndrome	1 (1.25)	5 (6.25)	–	–
arrhythmia	2 (2.50)	7 (8.75)	–	–
Renal impairment	1 (1.25)	6 (7.50)	–	–
None	76 (95.00)	62 (77.50)	–	–
Total incidence	4 (5.00)	18 (22.50)	10.329	0.001

## Discussion

Cardiac surgery patients often present in a critical condition, with disease progression occurring rapidly and necessitating prompt surgical intervention. During such surgeries, the heart endures prolonged ischemia and reperfusion injury, which can impair cardiac contraction and relaxation, leading to acute heart failure [7, 10]. This condition manifests clinically as dyspnea, fatigue, dizziness, palpitations, chest congestion, shortness of breath, decreased blood pressure, increased heart rate, and arrhythmias, all of which adversely affect patient prognosis [1, 11]. Effective monitoring of cardiac function is crucial in critically ill patients undergoing cardiac surgery to prevent and manage acute heart failure early.

Current treatment strategies for acute heart failure predominantly involve the use of positive inotropic drugs such as dopamine, dobutamine, and epinephrine, which significantly enhance cardiac function [16, 17]. These drugs work by stimulating *β*-receptors, leading to increased myocardial contractility, vasodilation, and reduced peripheral resistance. Dopamine and dobutamine improve hemodynamics by dilating renal, coronary, and cerebral vessels [4, 30]. Adrenaline, through *α* and *β* receptor stimulation, enhances coronary vessel dilation and improves heart function (Lympelopoulos et al., 2021 [26]). However, the increased myocardial contractility associated with these agents can result in calcium imbalance, elevated intracellular calcium levels, reduced tissue ATP, increased cardiac oxygen consumption, and a higher risk of arrhythmias and myocardial damage, which can exacerbate the condition and elevate patient mortality [18].

Levosimendan, a newer cardiac stimulant, offers a promising alternative. It enhances myocardial contractility by sensitizing cardiac myofilaments to calcium, without increasing intracellular calcium levels, thus minimizing the risk of arrhythmias and myocardial oxygen consumption [2, 29]. Additionally, levosimendan improves ventricular diastolic function and reduces cardiac stress by its vasodilatory effects, which collectively contribute to better management of acute heart failure [8].

In our study, patients treated with levosimendan exhibited significant improvements in hemodynamic parameters, including decreased central venous pressure, heart rate, and lactic acid levels, and increased systolic blood pressure and urine output. This suggests that levosimendan enhances cardiac function by promoting effective Ca2 + binding to troponin C, thereby improving myocardial contraction and overall hemodynamic stability. Additionally, we observed improvements in left ventricular function, with increased left ventricular ejection fraction (LVEF) and decreased left ventricular end-diastolic volume index (LVEDVi) and left ventricular end-systolic volume index (LVESVi). These changes indicate enhanced cardiac contractile function and reduced ventricular stress [20, 22, 28].

NT-Pro-BNP, a biomarker of heart failure severity and ventricular dysfunction, was significantly lower in the research group compared to the control group, further supporting the effectiveness of levosimendan in improving cardiac function [24]. The lower incidence of complications, including arrhythmias and renal impairment, in the research group highlights the safety and efficacy of levosimendan in reducing adverse outcomes associated with acute heart failure.

In conclusion, patients undergoing cardiac surgery are highly susceptible to acute heart failure, which can significantly impact recovery and outcomes. Levosimendan therapy proves to be a valuable intervention, offering substantial improvements in cardiac function and hemodynamic stability while reducing the incidence of complications. Its unique mechanism of action and favorable safety profile make it an attractive option for clinical use in managing acute postoperative heart failure.

## Data Availability

The datasets used or analyzed during the current study are available from the corresponding author upon reasonable request.
